# Fatigue, Tiredness, Lack of Energy, and Sleepiness in Multiple Sclerosis Patients Referred for Clinical Polysomnography

**DOI:** 10.1155/2012/673936

**Published:** 2012-12-20

**Authors:** Tiffany J. Braley, Ronald D. Chervin, Benjamin M. Segal

**Affiliations:** ^1^Department of Neurology, Sleep Disorders Center, and Holtom-Garrett Program in Neuroimmunology, University of Michigan, C728 Med-Inn Building, 1500 E. Medical Center Drive, Ann Arbor, MI 48109, USA; ^2^Department of Neurology and Sleep Disorders Center, University of Michigan, C728 Med-Inn Building, 1500 E. Medical Center Drive, Ann Arbor, MI 48109, USA; ^3^Department of Neurology and Holtom-Garrett Program in Neuroimmunology, University of Michigan, 4013 Biomedical Science Research Center, 109 Zina Pitcher Place, SPC 2200, Ann Arbor, MI 48109, USA

## Abstract

*Objectives*. To assess the relationship between nocturnal polysomnographic (PSG) findings and a group of key self-reported symptoms—fatigue, tiredness, lack of energy, and sleepiness—among sleep-laboratory referred patients with and without multiple sclerosis (MS). *Methods*. PSG and questionnaire data from *n* = 30 MS patients and *n* = 30 matched controls were analyzed retrospectively. Associations between symptoms of fatigue, tiredness, lack of energy, sleepiness, and PSG variables of interest were examined among MS patients and controls. *Results*. More MS patients than controls reported fatigue, tiredness, and lack of energy to occur often or almost always (Chi-square *P* < 0.0001 for each), but sleepiness was reported similarly by both groups (*P* = 0.3409). Among MS patients, tiredness correlated with sleepiness (Spearman correlation *P* = 0.005), and a trend emerged toward correlation between fatigue and sleepiness (Spearman correlation *P* = 0.076). Decreased sleep efficiency on PSGs correlated with fatigue, tiredness, and lack of energy in MS patients (Spearman correlation *P* = 0.002, 0.029, and 0.048, resp.), but not sleepiness or any symptom among controls. *Conclusion*. In comparison to controls, MS patients report more fatigue, tiredness, and lack energy, but not sleepiness. Fatigue and related symptoms may arise from MS itself or in relation to reduced sleep efficiency.

## 1. Introduction

Multiple sclerosis (MS) is an autoimmune disease of the central nervous system that causes myelin destruction and axonal damage in the brain and spinal cord. It is the leading cause of nontraumatic neurological disability among young adults and is associated with a variety of debilitating symptoms, including fatigue. 

Fatigue is the most common symptom experienced by persons with MS, affecting up to 90% of patients at some point in their disease course [1–3]. Fatigue imposes significant socioeconomic consequences, including loss of work hours and employment [4], and is a prominent cause of diminished quality of life among individuals with MS [3]. Despite its prevalence in MS as well as other medical conditions, there is no unified definition for fatigue. Consequently, there is potential for considerable overlap between fatigue and other subjective terms commonly used by MS patients to describe lack of energy or alertness, including sleepiness.

Sleep disorders are traditionally recognized for their contributions to excessive daytime sleepiness. However, many subjects in the general population who have sleep disorders such as obstructive sleep apnea report that problems with fatigue, tiredness, or lack of energy supersede problems with sleepiness [5] and experience improvement in these symptoms when their apnea is treated [6]. Nonetheless, little is known about the most common symptoms experienced by MS patients with obstructive sleep apnea and other sleep disorders, or ways in which MS patients may differ from non-MS patients in how they describe symptoms that could be attributable to sleep disorders. Research to clarify the relationship between sleep disorders, fatigue, and related symptoms in MS could help clinicians identify which MS patients are most likely to benefit from sleep evaluations and facilitate early treatment for common underlying sleep disorders. The purpose of this study was to assess the relationship between polysomnographic findings and the frequency of self-reported daytime symptoms (fatigue, tiredness, lack of energy, and sleepiness), among MS patients referred for clinical polysomnography (PSG), compared to referred controls without MS.

## 2. Methods

This retrospective data analysis was approved by the University of Michigan Institutional Review Board.

### 2.1. Subjects/Data Collection

#### 2.1.1. MS Cases

 Subject selection and data collection methods have been described previously [7]. In summary, demographic, clinical, and polysomnographic (PSG) data were assembled from the University of Michigan (U-M) Sleep Disorders Center database and medical records for *n* = 48 patients, 18 years or older, who had an established diagnosis of MS based on the McDonald diagnostic criteria and had completed a clinical overnight PSG between March 1999 and June 2010. Only patients who had completed the University of Michigan Sleepiness Impact Assessment (UMSIA) at the time of PSG (*n* = 30) were considered eligible for the analyses.

#### 2.1.2. Controls

 Control subjects without MS (*n* = 30) who had also completed at least 2 out of 4 categorical responses of interest on the UMSIA were selected from more than 8,000 adult patients referred for diagnostic sleep studies. Controls were matched to each MS patient in a 1 : 1 ratio for paired analyses, based on age (±5 years), gender, and body mass index (±2 kg/m^2^). Matched controls were also selected based on date of study, after or before January 1st, 2008, to control for minor changes in PSG scoring criteria that took place following January 1st, 2008 [8].

#### 2.1.3. Data Collection

 The following variables were extracted from the sleep database: PSG date, gender, age, body mass index (BMI), PSG diagnosis, apnea-hypopnea index (AHI or rate of apneas and hypopneas per hour of sleep), percentage of sleep time spent in stage 1 sleep (*N*1%), percentage in stage 2 sleep (*N*2%), percentage in stage 3 (slow wave) sleep (*N*3%), percentage in REM sleep (REM%), arousal index (number of arousals per hour of sleep), number of sleep stage shifts, sleep latency, sleep efficiency (SE, ratio of time spent asleep to the amount of time spent in bed), the periodic leg movement index (PLMI), and Epworth Sleepiness Scale (ESS) scores. The Epworth Sleepiness Scale is an eight-item questionnaire that asks the patient to rate, on a Likert scale, the likelihood of dozing in a variety of sedentary situations [9]. Medical records were reviewed to confirm eligibility and extract additional data regarding MS-specific variables. Individuals with concomitant diseases that could increase the risk of sleep apnea or influence fatigue level, including cancer, severe cardiopulmonary disease, pregnancy, major depressive disorder (within 6 months of PSG), or neurologic diseases other than MS, were excluded. For MS subjects, additional variables recorded included MS subtype (relapsing-remitting versus progressive), disease duration at time of PSG (years), use of disease modifying therapy (DMT, defined as glatiramer acetate or beta-interferon use at the time of PSG), and estimates of disability (defined as an EDSS score of less than 6.0 (low disability) or greater than or equal to 6.0 (high disability)) [10].

### 2.2. The University of Michigan Sleepiness Impact Assessment

Developed in 1996 by one of the investigators (RC), the University of Michigan Sleepiness Impact Assessment (UMSIA) is a self-administered questionnaire that allows patients to rate, in a parallel manner, the frequency that sleepiness and associated symptoms affect their daily life using 5-point Likert scales [5]. For our study, categorical responses regarding the frequency of problematic sleepiness, fatigue, tiredness, and lack of energy (rated as “never” = 1, “seldom” = 2, “occasionally” = 3, “often” = 4, and “almost always” = 5) were used for the analyses. 

### 2.3. Polysomnography

Full, laboratory-based polysomnography (PSG) and scoring followed existing standards before 2008 [11], and then slightly different, newly published standards from that point forward [8]. The main relevant change concerned use of nasal pressure to identify hypopneas and rules employed to score them. Our laboratory had already been using thoracic or abdominal excursion changes, in addition to thermocouple airflow changes (when any of these were followed by awakenings, arousals, or ≥4% oxygen desaturations) to identify hypopneas in a sensitive manner before the AASM 2007 standards were published. The apnea-hypopnea index (AHI) was calculated as the number of obstructive apneas, central apneas, or hypopneas per hour of sleep. The presence of obstructive sleep apnea was defined by an apnea-hypopnea index of at least five episodes per hour of sleep [12]. Sleep stages were scored according to standard criteria [8, 11]. 

### 2.4. Statistical Methods

UMSIA responses for each symptom (fatigue, tiredness, lack of energy, and sleepiness) were dichotomized into 2 categories: frequent (for responses of “often” or “almost always”) and infrequent (for responses of “never,” “seldom,” or “occasionally”) and compared between MS patients and controls using Chi-square tests.

Two-sample *T* tests (for normal continuous data), Chi-square tests (for dichotomized data), and Wilcoxon rank-sum tests (for nonnormal continuous data) were used to compare baseline characteristics among MS patients and controls. Spearman correlation tests were used to examine associations between categorical UMSIA responses (reported frequencies of problematic fatigue, tiredness, lack of energy, and sleepiness), Epworth scores, demographic information, and PSG parameters of interest (AHI, sleep efficiency, *N*1%, *N*2%, *N*3%, REM%, sleep latency, arousal index, number of sleep stage shifts, and PLMI) separately within MS and control groups. 

Chi-square tests, *T* tests, and multiple linear regression models were also used to compare dichotomized UMSIA responses and PSG variables between MS subgroups of interest (relapsing-remitting versus progressive MS, use of DMT and disability status, as defined in Section 2.1.3). 

Statistical tests were performed using SAS version 9.2. Tests were two sided with level of statistical significance set at 0.05.

## 3. Results

### 3.1. Baseline Data

 Baseline characteristics for *n* = 30 MS patients and *n* = 30 matched controls are shown in Table 1. No significant differences between MS subjects and matched controls emerged for matched variables (BMI, gender, or age) or the other comparators listed, except for number of sleep stage shifts (*P* = 0.017). For MS patients, mean disease duration was 12.0 years. Seventy percent of MS subjects and seventy percent of matched controls were female, consistent with the gender distributions seen in MS in the United States. For those with available data, 21 (70%) MS subjects had relapsing-remitting disease, and nine (30%) had progressive disease. Twenty (67%) of the MS patients were on disease modifying therapy (DMT), and ten (33%) were not. Eighty percent of MS patients and 63% of controls met diagnostic criteria for obstructive sleep apnea (see Section 2.3). 

### 3.2. MS Patients versus Controls

**Figure 1:**
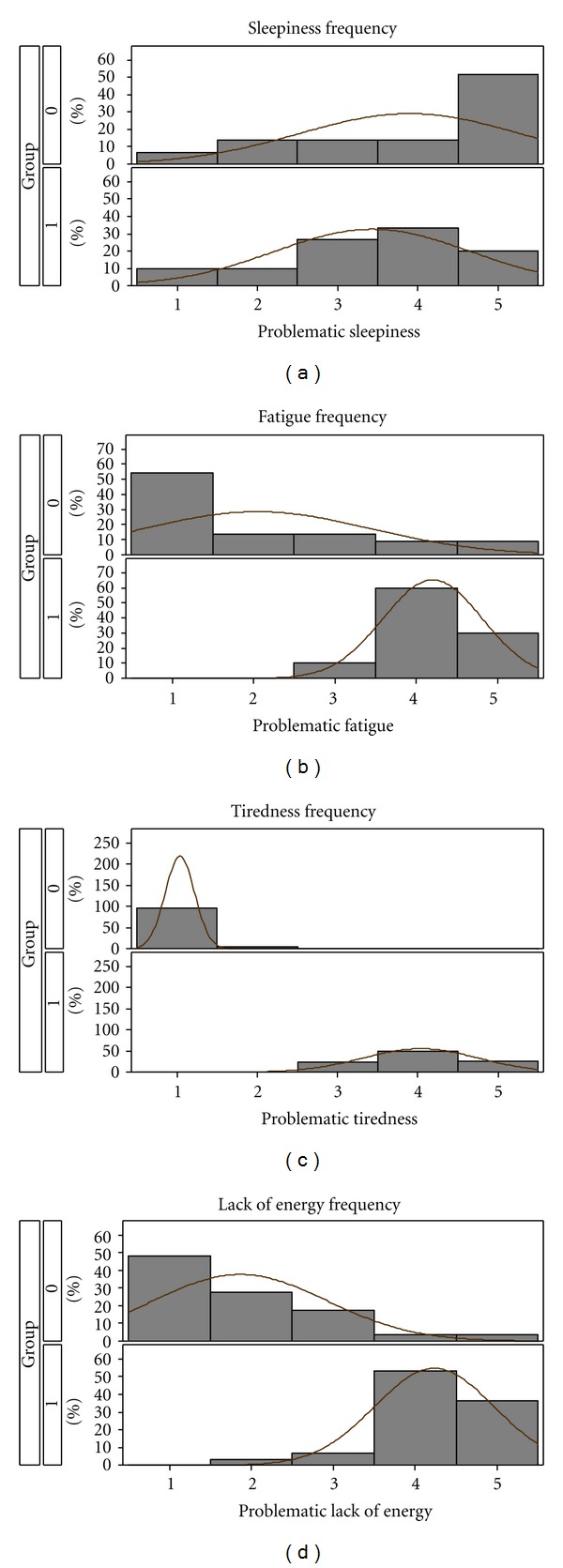
Frequencies of Likert responses for problematic sleepiness (a), fatigue (b), tiredness (c), and lack of energy (d) among MS patients (group 1) and matched controls (group 0). 1 = “never;” 2 = “seldom;” 3 = “occasionally;” 4 = “often;” and 5 = “almost always”.

 Response rates to categorical UMSIA items and distribution of symptom frequencies for MS patients and matched controls are shown in Figure 1. When responses were dichotomized, significantly more MS patients than controls reported fatigue, tiredness, and lack of energy to occur often or almost always (90% versus 18%, 77% versus 0%, and 90% versus 7%; Chi-square *P* < 0.0001 for each). Conversely, frequent sleepiness as per the UMSIA was reported similarly by both groups (53% versus 65%; *P* = 0.3409). There were strong correlations between fatigue, tiredness, and lack of energy in both groups (Table 2). Among MS patients, tiredness correlated with sleepiness, (rho = 0.50; *P* = 0.005), and a trend emerged toward a significant correlation between fatigue and sleepiness (rho = 0.33; *P* = 0.076). Among controls, fatigue correlated with sleepiness (rho = 0.45; *P* = 0.034).

Decreased nocturnal sleep efficiency correlated with fatigue, tiredness, and lack of energy in MS patients (rho = −0.55, *P* = 0.002; rho = −0.40, *P* = 0.029; and rho = −0.36, *P* = 0.048, resp.), but not with sleepiness in MS patients or with any symptom among controls. The apnea-hypopnea index was not associated with increased frequency of any symptom. Mean Epworth Sleepiness Scale scores did not significantly differ among groups. No significant relationship emerged between mean Epworth scores and UMSIA reports of sleepiness among MS patients or controls, although trends suggested a positive correlation between Epworth score and fatigue, and between ESS score and tiredness in the MS group (Table 2). 

A trend toward a significant increase in AHI among MS patients compared to controls emerged in regression models, after adjustment for age and BMI (*P* = 0.0647, not shown). No significant difference was noted in mean *N*1%, *N*3%, REM%, arousal index, sleep latency, or sleep efficiency among MS patients compared to controls, though a trend toward decreased *N*2% emerged in the MS group. There was also a nonsignificant increase in mean PLMI among MS patients compared to controls. 

### 3.3. MS-Specific Analyses

 Dichotomized frequencies of fatigue, tiredness, lack of energy, and sleepiness (often or always versus less commonly) did not significantly vary by MS subtype or disability status. Sleep efficiency and REM% were significantly decreased in progressive MS subtypes compared to relapsing-remitting MS, after adjusting for AHI and age in regression models (*P* = 0.0442 and 0.0373, resp.) and in subjects with high disability (EDSS ≥ 6.0) compared to those with low (EDSS < 6.0) disability status (*P* = 0.0048 and 0.0061, resp.). The association between progressive MS and apnea severity (AHI) approached significance in regression models, after adjustment for age and BMI (*P* = 0.0596). The PLMI did not vary by MS subtype or disability status.

The presence or absence of disease modifying therapy (DMT) also did not influence the frequency of any symptom. Epworth scores, sleep efficiency, sleep stages, and the PLMI were not influenced by DMT use, but use of DMT was associated with decreased AHI (*T*-test *P* = 0.0419). Details regarding the relationships between DMT, disease subtype, and AHI in all *n* = 48 MS subjects with available PSG data (regardless of UMSIA completion), after adjustment for additional variables known to influence AHI, have been described previously [7] (see Section 2.1).

## 4. Discussion

 This study of symptoms and PSG findings among MS patients and non-MS controls shows that MS patients with suspected sleep problems are more likely than controls to emphasize problematic fatigue, tiredness, and lack of energy, as opposed to sleepiness. Furthermore, decreased sleep efficiency correlated with increased symptoms of tiredness, fatigue, and lack of energy in patients with MS. In contrast, sleep efficiency did not correlate with any such symptom among controls. Our findings are particularly surprising given that sleep efficiency itself, objectively assessed on polysomnography, was essentially identical between MS patients and controls. These data raise important considerations for understanding fatigue and related symptoms among persons with MS. In particular, our results suggest that patients with MS may exhibit an increased sensitivity to the downstream effects of diminished sleep efficiency compared to patients without MS. Our findings raise the possibility that measures to increase sleep efficiency in MS could improve daytime fatigue, regardless of whether concomitant sleep disorders are present.

 While fatigue has long been recognized for its impact on the health and quality of life of persons with MS, separation of treatable from untreatable causes can be challenging, given the wide range of potential etiologies. In addition to contributions from cytokine dysregulation, MS-related fatigue may also be influenced by CNS lesion burden, medication effects, and other conditions concomitant with MS, including depression, pain, and sleep disturbances. The lack of a unified definition for fatigue further contributes to its complexity. While validated instruments to quantify fatigue or assess its impact on the function of persons with MS are commonly employed in both clinical and research settings, most of these instruments do not include items to define fatigue or describe it in the context of similar symptoms such as sleepiness. Subsequently, subjects are left to quantify their fatigue on the basis of their own subjective interpretation, which may vary by education, disease-specific biases, or cultural backgrounds. This ambiguity increases the potential for overlap among terms used to describe symptoms experienced by MS patients with concomitant sleep disorders. Consequently, MS patients suffering from sleep disorders who describe symptoms other than sleepiness may escape formal sleep evaluations, as fatigue and related symptoms are expected consequences of MS itself.

Growing evidence suggests that sleep disturbances may be closely linked to fatigue in persons with MS. A recent prospective PSG study in MS subjects and controls suggests that severe obstructive sleep apnea (defined as an AHI ≥ 30) is independently associated with fatigue in MS, as defined by the Fatigue Severity Scale [13]. The nine-response Fatigue Severity Scale (FSS) assesses the impact of fatigue on multiple outcomes, with a physical focus, using a 7-point Likert scale [14]. 

A separate group [15] found that fatigue, quantified using a French version of the Fatigue Impact Scale (FIS) [16], correlated strongly with sleep disturbances as measured by the Pittsburgh Sleep Quality Index among individuals with MS [17]. The FIS reflects functional limitation due to fatigue experienced within the previous month. A similar association was also demonstrated with the FSS as the primary fatigue outcome measure [18]. Using home PSG, Veauthier and colleagues reported an increase in the prevalence of clinically relevant sleep disorders, including sleep disordered breathing [19], in fatigued MS patients compared to nonfatigued patients as defined by the Modified Fatigue Impact Scale. 

Whereas the above studies highlight fatigue as a potential consequence of sleep disturbances in MS, these and other studies of relationships between fatigue and sleep disorders in MS have relied on instruments that do not allow a direct comparison between sleepiness, fatigue, and related symptoms in a systematic manner. Our study is the first to address each of these symptoms separately using parallel phrasing, while relating them to objective PSG measures [5]. While the UMSIA questionnaire has yet to be validated in patients with MS, our data suggest that MS patients with sleep disorders may share a propensity for terms other than sleepiness to describe their symptoms. Furthermore, use of these terms may signal decreased sleep efficiency in this population.

One possible explanation for the strong correlation between fatigue level and sleep efficiency in MS may relate to what is known about the role of cytokine dysregulation in fatigue. Proinflammatory cytokines, produced by autoimmune effector cells, are proposed to contribute to fatigue in MS [20, 21]. It is noteworthy that two of these cytokines, IL-6 and TNF-alpha, are also elevated in the serum of individuals with obstructive sleep apnea and sleep deprivation and correlate with fatigue and sleepiness in general population studies [22–25]. While conclusions about a causal relationship between cytokine dysregulation and reported symptoms cannot be drawn from our retrospective cross-sectional study, our findings highlight a common immunological pathway between MS and sleep disturbances, which may synergistically amplify fatigue when sleep efficiency is reduced. 

While the lack of correlation between sleepiness and sleep efficiency in both groups may appear counterintuitive, it is consistent with previous reports. Despite the traditional clinician emphasis on sleepiness, many subjects in the general population who have sleep disorders prefer to use terms other than sleepiness to describe their daytime symptoms [5]. Furthermore, previous data from our group have shown little or no association between measured sleep efficiency and either subjective [26] or objective [27] daytime sleepiness. Our current data support these findings.

Our study has some limitations. As it was retrospective, data on potential variables that could influence fatigue and related symptoms in the MS group were not available in a consistent format that could be useful in the analyses. These include detailed information on depressive symptoms, disease coping strategies, psychosocial factors, and medications (such as antispasmodics, narcotics, or sleep aids) that are often used by patients with MS, as well as MRI lesion burden and respiratory function, which could influence apnea scores. Subjects whose records indicated severe depression in medical charts were excluded from analyses, but milder depressive symptoms that may not warrant a diagnosis of major depression could not be reliably studied from this retrospective chart review. Finally, the number of completed UMSIA responses for fatigue frequency was by chance slightly lower in the control group, which could theoretically explain the differences in correlation between fatigue level and sleep efficiency among MS patients versus controls. This is thought to be less likely, however, given the strong differences in correlation between tiredness/lack of energy and sleep efficiency among MS patients versus controls, where the number of completed UMSIA responses was similar for both groups. 

Despite these limitations, our data suggest that MS patients referred to a sleep laboratory are more likely than referred controls to complain of fatigue and related symptoms other than sleepiness, a more widely recognized consequence of sleep disturbances. Our study highlights the importance of considering physiologic sleep disturbance in MS patients who complain of fatigue, tiredness, lack of energy, or sleepiness.

## 5. Conclusion

Our data suggest that MS patients, in comparison to matched controls also referred for polysomnography, report more fatigue, tiredness, and lack energy, but not sleepiness. Fatigue and significant symptoms other than sleepiness may arise from MS itself or in relation to sleep disturbance as reflected by reduced sleep efficiency in particular, which could offer opportunities for effective intervention.

## Figures and Tables

**Table 1:** Baseline characteristics, polysomnographic findings, and results of univariate analyses for MS patients compared to matched controls. *P value <0.05.

	MS (*n* = 30)	Controls (*n* = 30)	*P* value
Age (years, mean ± SD)	46.7 (11.3)	45.9 (10.7)	0.762
Gender (female %)	70%	70%	1.0
Body mass index (kg/m^2^, mean ± SD)	32.2 (4.5)	32.3 (4.9)	0.999
Apnea-hypopnea index	14.5 (12.1)	9.6 (8.6)	0.078
Obstructive sleep apnea (%)	80%	63.3%	0.152
Percentage of time spent in stage 1 sleep (mean *N*3%, ± SD)	18.8 (13.5)	13.7 (7.0)	0.074
Percentage of time spent in stage 2 sleep (mean *N*3%, ± SD)	56.0 (13.2)	62.3 (12.2)	0.059
Percentage of time spent in slow wave sleep (mean *N*3%, ± SD)	10.8 (9.2)	7.3 (7.3)	0.106
Percentage of time spent in REM sleep (mean REM%, ± SD)	14.4 (8.1)	16.7 (7.5)	0.251
Sleep efficiency (mean ± SD)	80.5 (12.0)	79.2 (13.6)	0.697
Arousal index (number of arousals per hour of sleep, mean ± SD)	18.7 (12.6)	17.2 (12.0)	0.689
Number sleep stage shifts (mean ± SD)	168.0 (68.2)	133.8 (31.9)	0.017*
Sleep latency, in minutes (mean ± SD)	24.8 (25.7)	21.9 (22.7)	0.641
Periodic leg movement index (mean ± SD)	16.0 (28.7)	6.9 (13.0)	0.122
MS disease duration (years, mean ± SD)	12.0 (10.8)	N/A	N/A
Disease-modifying therapy (%)	66.7%		
Expanded disability status scale (% ≥6.0)	30.0%		
Relapsing-remitting MS (%)	70.0%		
Progressive MS (%)	30.0%		

**Table 2:** Spearman correlation test results for University of Michigan Sleepiness Impact Assessment responses and polysomnographic parameters in MS patients (top) and matched controls (bottom). Values listed as Spearman coefficient (*P* value).

MS patients						
	Fatigue	Tiredness	Lack of energy	Sleepiness	Sleep efficiency	Epworth sleepiness scale
Fatigue	1.0	0.77 (<0.0001)	0.56 (<0.0001)	0.33 (0.076)	−0.55 (0.002)	0.36 (0.073)
Tiredness		1.0	0.42 (0.020)	0.50 (0.005)	−0.40 (0.029)	0.370 (0.063)
Lack of energy			1.0	0.25 (0.180)	−0.36 (0.048)	0.29 (0.147)
Sleepiness				1.0	−0.13 (0.507)	0.24 (0.245)

Matched controls						

Fatigue	1.0	0.38 (0.0838)	0.47 (0.028)	0.45 (0.034)	−0.05 (0.833)	0.22 (0.350)
Tiredness		1.0	0.24 (0.204)	0.17 (0.375)	0.29 (0.121)	0.05 (0.802)
Lack of energy			1.0	0.20 (0.309)	−0.17 (0.389)	0.11 (0.580)
Sleepiness				1.0	0.30 (0.116)	0.22 (0.282)

## References

[1] Krupp L (2006). Fatigue is intrinsic to multiple sclerosis (MS) and is the most commonly reported symptom of the disease. *Multiple Sclerosis*.

[2] Lerdal A, Celius EG, Krupp L, Dahl AA (2007). A prospective study of patterns of fatigue in multiple sclerosis. *European Journal of Neurology*.

[3] Janardhan V, Bakshi R (2002). Quality of life in patients with multiple sclerosis: the impact of fatigue and depression. *Journal of the Neurological Sciences*.

[4] Smith MM, Arnett PA (2005). Factors related to employment status changes in individuals with multiple sclerosis. *Multiple Sclerosis*.

[5] Chervin RD (2000). Sleepiness, fatigue, tiredness, and lack of energy in obstructive sleep apnea. *Chest*.

[6] Chotinaiwattarakul W, O’Brien LM, Fan L, Chervin RD (2009). Fatigue, tiredness, and lack of energy improve with treatment for OSA. *Journal of Clinical Sleep Medicine*.

[7] Braley TJ, Segal BM, Chervin RD (2012). Sleep-disordered breathing in multiple sclerosis. *Neurology*.

[8] Iber C, American Academy of Sleep Medicine (2007). *The AASM Manual for the Scoring of Sleep and Associated Events: Rules, Terminology and Technical Specifications*.

[9] Johns MW (1991). A new method for measuring daytime sleepiness: the Epworth sleepiness scale. *Sleep*.

[10] Kurtzke JF (1983). Rating neurologic impairment in multiple sclerosis: an expanded disability status scale (EDSS). *Neurology*.

[11] Rechtshaffen A, Kales A (1968). *A Manual of Standardized Terminology, Techniques and Scoring System for Sleep Stages of Human Subjects*.

[12] (1999). Sleep-related breathing disorders in adults: recommendations for syndrome definition and measurement techniques in clinical research. The report of an American Academy of Sleep Medicine Task Force. *Sleep*.

[13] Kaminska M, Kimoff R, Benedetti A (2012). Obstructive sleep apnea is associated with fatigue in multiple sclerosis. *Multiple Sclerosis*.

[14] Krupp LB, LaRocca NG, Muir-Nash J, Steinberg AD (1989). The fatigue severity scale. Application to patients with multiple sclerosis and systemic lupus erythematosus. *Archives of Neurology*.

[15] Neau JP, Paquereau J, Auche V (2012). Sleep disorders and multiple sclerosis: a clinical and polysomnography study. *European Neurology*.

[16] Fisk JD, Pontefract A, Ritvo PG, Archibald CJ, Murray TJ (1994). The impact of fatigue on patients with multiple sclerosis. *Canadian Journal of Neurological Sciences*.

[17] Buysse DJ, Reynolds CF, Monk TH, Berman SR, Kupfer DJ (1989). The Pittsburgh sleep quality index: a new instrument for psychiatric practice and research. *Psychiatry Research*.

[18] Kaynak H, Altintaş A, Kaynak D (2006). Fatigue and sleep disturbance in multiple sclerosis. *European Journal of Neurology*.

[19] Veauthier C, Radbruch H, Gaede G (2011). Fatigue in multiple sclerosis is closely related to sleep disorders: a polysomnographic cross-sectional study. *Multiple Sclerosis*.

[20] Heesen C, Nawrath L, Reich C, Bauer N, Schulz KH, Gold SM (2006). Fatigue in multiple sclerosis: an example of cytokine mediated sickness behaviour?. *Journal of Neurology, Neurosurgery and Psychiatry*.

[21] Flachenecker P, Bihler I, Weber F, Gottschalk M, Toyka KV, Rieckmann P (2004). Cytokine mRNA expression in patients with multiple sclerosis and fatigue. *Multiple Sclerosis*.

[22] Vgontzas AN, Zoumakis E, Lin HM, Bixler EO, Trakada G, Chrousos GP (2004). Marked decrease in sleepiness in patients with sleep apnea by etanercept, a tumor necrosis factor-*α* antagonist. *Journal of Clinical Endocrinology and Metabolism*.

[23] Vgontzas AN, Bixler EO, Lin HM, Prolo P, Trakada G, Chrousos GP (2005). IL-6 and its circadian secretion in humans. *NeuroImmunoModulation*.

[24] Hegglin A, Schoch OD, Korte W, Hahn K, Hürny C, Münzer T (2012). Eight months of continuous positive airway pressure (CPAP) decrease tumor necrosis factor alpha (TNFA) in men with obstructive sleep apnea syndrome. *Sleep and Breathing*.

[25] Steiropoulos P, Papanas N, Nena E (2010). Inflammatory markers in middle-aged obese subjects: does obstructive sleep apnea syndrome play a role?. *Mediators of Inflammation*.

[26] Chervin RD, Aldrich MS, Pickett R, Guilleminault C (1997). Comparison of the results of the Epworth sleepiness scale and the multiple sleep latency test. *Journal of Psychosomatic Research*.

[27] Chervin RD, Kraemer HC, Guilleminault C (1995). Correlates of sleep latency on the multiple sleep latency test in a clinical population. *Electroencephalography and Clinical Neurophysiology*.

